# Enteric Duplication Cyst Containing Squamous and Respiratory Epithelium: An Interesting Case of a Typically Pediatric Entity Presenting in an Adult Patient

**DOI:** 10.1155/2014/790326

**Published:** 2014-08-20

**Authors:** Jessica Leigh Baumann, Charmi Patel

**Affiliations:** Department of Pathology, University of Arizona, 1501 N. Campbell Avenue, P.O. Box 245108, Tucson, AZ 85724-5108, USA

## Abstract

Enteric duplication cysts are rare congenital malformations that can occur at any point along the digestive tract, most commonly the small bowel. They are characterized by the presence of an outer layer of smooth muscle and an inner lining of mucosa that may resemble any portion of the digestive tract. Less commonly, cases have been reported that also contain mucosal components of nonintestinal origin. This entity is typically diagnosed in young children, but occasionally presents in adolescence and young adulthood. We present a rare case of a 21-year-old male who presented with nonspecific symptoms of abdominal discomfort and weight loss and was later found to have a 9 cm nonenhancing mass in the distal ileum on CT imaging. Laparoscopic dissection of the mass revealed a cystic lesion lined mainly by pseudostratified ciliated columnar respiratory-type epithelium, with patchy areas of squamous epithelium as well as villous columnar epithelium resembling small bowel. The unique histology and advanced patient age make this case a unique presentation of what is already a rare pathological entity.

## 1. Introduction

Enteric duplication cysts are rare congenital malformations that can occur at any point along the digestive tract, most typically on the mesenteric side of the small bowel [1]. The epithelial lining of the cysts resembles that of some element of the digestive tract, though the exact type varies from case to case [1]. Duplication cysts can occur in the context of other congenital abnormalities, such as those of the vertebral bodies and genitourinary tract, though they most often are seen in patients without other developmental malformations [2–4]. The lesions are typically present in the first year of life (70% of cases) or early childhood, though they more rarely have been reported in older patients [5–7]. Initial symptomatology is dictated by the size and location of the lesion and most commonly includes obstruction, with or without vomiting, abdominal distension, dysphagia, palpable mass, or, more infrequently, urinary symptoms [4, 5, 8]. We herein present a unique case of a young adult patient with an enteric duplication cyst lined by both respiratory and squamous epithelia, the latter of which we have only rarely seen previously reported in this clinical entity.

## 2. Case Presentation

A previously healthy 21-year-old male presented with a 3-month history of crampy epigastric pain, diarrhea, and persistent nausea. Prior to the onset of his GI symptoms, his only past medical issue had been asthma, and other than occasional alcohol and tobacco use, he did not have any history of significant occupational exposure or abuse of elicit substances. Over the time course of his abdominal symptoms, the patient reported a 30–40 pound unintentional weight loss. He denied any accompanying fevers and chills, melena, hematochezia, hematemesis, or dysuria. The severity of the pain had led him to seek emergency room evaluation once before, at which time he was treated with analgesics and was sent home after a negative abdominal scan. A CT scan of the abdomen/pelvis subsequently ordered by the patient's primary care provider revealed a well-circumscribed 4.5 cm × 9 cm nonenhancing oval mass in the ileocecal region, appearing separated from the urinary bladder and sigmoid colon, but in communication with the small bowel. Differential diagnosis based on imaging included a small bowel lymphoma versus gastrointestinal stromal tumor (GIST). He was referred to our center for surgical intervention. The decision was made to proceed with a diagnostic laparoscopy with pelvic mass resection and possible small bowel resection.

Laparoscopy revealed a fluctuant cystic lesion measuring 6.4 cm × 5.9 cm × 4.6 cm involving the ileal portion of the small bowel, approximately 10 cm proximal to the ileocecal valve. Terminal ileal resection with right hemicolectomy was performed. Dissection of the cystic cavity in the terminal ileum revealed a smooth and glistening lining, and the lesion was filled with a tan and gelatinous material. The cyst was not in communication with the lumen of the ileum. The remainder of the specimen, including the ascending colon, the appendix, and the rest of the resected ileum, was unremarkable. The pelvis was otherwise benign, smooth, and without peritoneal lesions.

**Figure 1:**
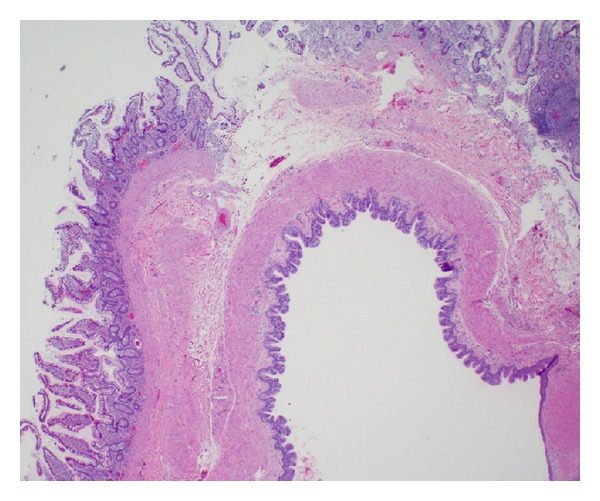
A representative section of cyst lining showing the presence of two distinct muscle layers. This section illustrates the variety of mucosal types found throughout the cyst cavity, including (from left to right) columnar epithelium with goblet cells, ciliated respiratory-like epithelium, and small portion of squamous epithelium (20x magnification).

**Figure 2:**
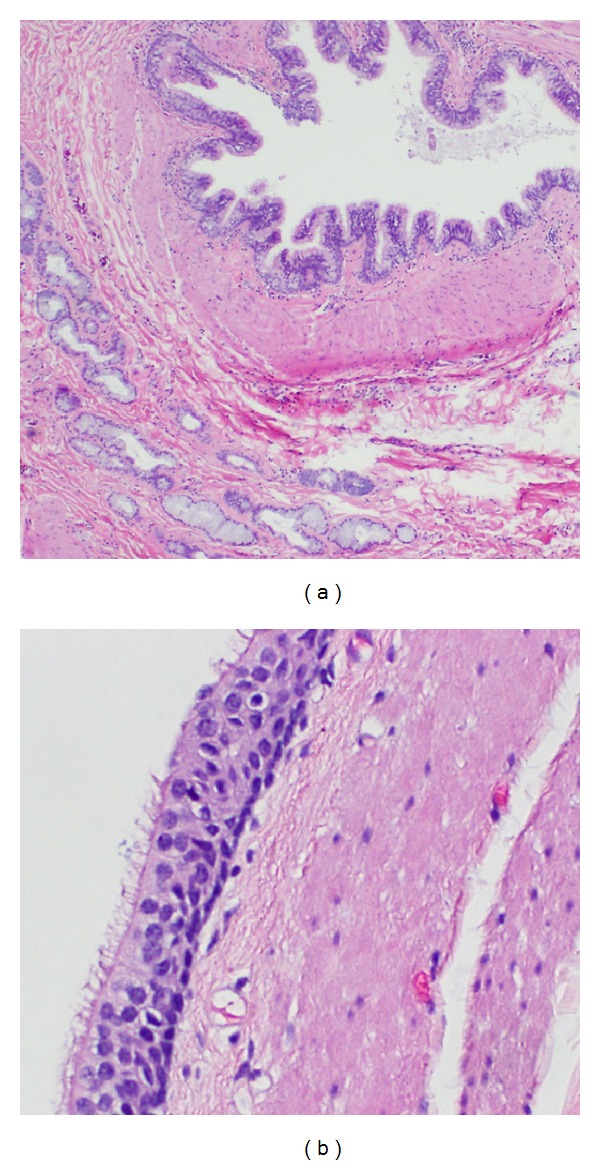
Area of cyst lining showing respiratory-like mucosa and underlying mucous glands ((a), 40x magnification). A higher-power magnification shows areas of mucosa with ciliated cells ((b), 400x).

**Figure 3:**
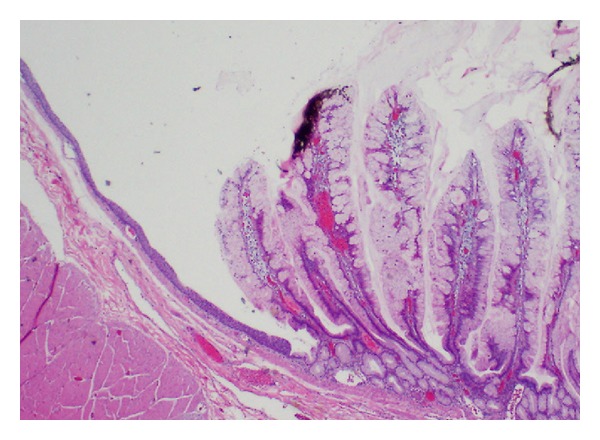
A sample of cyst lining showing an abrupt transition between squamous epithelium (left) and villous epithelium (40x magnification).

Microscopic evaluation of the lesion revealed that the cyst was lined mainly by pseudostratified ciliated columnar respiratory-type epithelium. There were also patchy mucosal areas lined by stratified squamous and small bowel type villous epithelium. Two distinct muscle layers were present. No malignancy or dysplasia was seen (Figure 1). In areas lined by respiratory type epithelium, mucous glands were present in the submucosa (Figure 2). An abrupt transition between the different epithelial types was easily appreciated (Figure 3). The appendix and segment of colon were without histopathologic abnormality. The findings were consistent with the appearance of an enteric duplication cyst. The patient's postoperative course was uneventful, and his subsequent abdominal MRI was unremarkable. He is symptom free and with no postsurgical complication 9 months after the procedure.

## 3. Discussion

Enteric duplication cysts are malformations of the digestive system, occurring most commonly in the small intestine, with the ileum representing up to 70% of cases [7]. By definition, cysts must contain at least a single outer muscular layer along with an inner lining of gastrointestinal mucosa, though cysts lined by multiple types of epithelia in addition to gastrointestinal epithelium have been reported [9]. They typically derive both their vascular supply and their smooth muscle wall from the adjacent intestinal segment, though they more rarely have an independent blood supply [1, 7]. Complications include perforation, intussusception, bowel obstruction from adjacent pressure or mass effect, volvulus, and associated malignancy [8]. While rare, malignancies arising within duplication cysts typically portend a poor prognosis, as most are diagnosed at advanced stages with widespread metastases. Reported cases have included squamous cell carcinoma, adenocarcinoma, and carcinoid tumors [7, 10]. In this case, the ileal location, nonspecific presenting abdominal symptomatology and absence of concomitant malignancy are consistent with the majority of cases of enteric cysts.

The exact etiology of these lesions is unknown, though proposed origins include a persistence of fetal enteric diverticula, intrauterine vascular occlusion, and failure of intestinal recanalization [6]. The last of these theories is perhaps the most favored and was originally proposed by Bremer in 1944. The transition of the embryological intestinal tract from a solid to a tubular state between 6 and 8 weeks of gestation involves the coalescence of multiple vacuoles, and derangement of this process may account for the formation of the double-walled enteric cysts [3, 11].

There are two main types of enteric duplication cysts, tubular and cystic, the latter one being the most common [10]. In 10–15% of cases, multiple cysts are detected concurrently [4]. The cysts may or may not communicate with adjacent bowel and typically will contain a translucent alkaline fluid. When ectopic gastric or pancreatic tissue is present, the fluid may alternatively be more acidic or contain pancreatic enzymes, respectively [12]. Those of the cystic type are typically lined by mucosa resembling the adjacent intestinal epithelium, while those of the tubular type may be lined by ectopic gastric mucosa [6]. Such lesions are more likely to be associated with cases that present with GI bleeding and can be detected via Tc-99m pertechnetate radionuclide scan, the same study used to detect ectopic gastric tissue in Meckel's diverticulum. However, as only about 35% of enteric duplication cysts will contain detectable gastric mucosa [13], isotope studies are of only limited diagnostic utility [5, 6].

Case reports have described the lining of cysts with tissue types representative of the entirety of the digestive tract, including pseudostratified columnar, gastric, cuboidal, or stratified squamous mucosa that may be of a disparate type to that found in adjacent normal tissue [14]. Other reported cases have included cysts at least partially composed of cellular components that do not resemble those naturally occurring in the GI tract, such as ciliated and respiratory-type epithelium and cartilage [8, 15]. This case falls in the minority of reported duplication cysts. In this lesion the lining of epithelium was composed of both squamous and respiratory mucosa, with little evidence of homology to the adjacent small bowel segment.

Detection and diagnosis of enteric cysts can be difficult, given that their symptomatology varies greatly and can mirror that of a wide spectrum of GI pathology. Imaging can aid in the narrowing of a wide range of differential diagnoses, especially amongst other unilocular cystic lesions, like those of the mesenteric, ovarian, choledochal, and hepatic subtypes [3]. Though conventional abdominal films rarely aid significantly in diagnosis, other imaging modalities, such as contrast studies, CT, or ultrasound (US), are helpful in the context of proper clinical correlation. US is currently the favored modality and will typically reveal a double-walled cyst appearing to have an echogenic inner mucosal layer and hypoechoic outer muscularis propria layer [5, 16]. The “double wall sign” can occasionally be mimicked by other GI or GU lesions, however, including Meckel's diverticulum, torsed ovarian cysts, and intestinal abscesses. Confirmation of imaging with characteristic histology is still required for definitive diagnosis [17]. CT imaging of enteric duplication cysts is somewhat less informative, as the mass will appear simply as a nonenhancing or slightly enhancing cystic lesion [1, 5], as noted in this case. Upper GI and barium contrast studies may sometimes reveal a filling defect or abnormal communication of a cystic structure with the bowel wall at the site of the mass [1, 6, 13].

The treatment of choice for enteric duplication cysts is complete surgical excision with reanastomosis [1]. The resection of the adjacent normal bowel wall is required due to the sharing of both a muscular wall and a vascular supply, though isolated removal of the cyst alone is possible in the rare cases that have an independent blood supply [18]. When the segment of affected bowel is too large to resect without risking short bowel syndrome, alternative treatment options include selective stripping of the affected intestinal mucosa [7, 19]. In patients with these anomalies an early accurate diagnosis is essential, because subsequent appropriate surgical treatment can lead to a satisfactory outcome.

## References

[1] Zahir I, Yusaf S, Zada F, Asif M, Akhtar N, Abbasi MZ (2010). Duplication cyst in a new born. *International Journal of Pathology*.

[2] Dutheil-Doco A, Le Pointe HD, Larroquet M, Ben Lagha N, Montagne J (1998). A case of perforated cystic duplication of the transverse colon. *Pediatric Radiology*.

[3] Teele RL, Henschke CI, Tapper D (1980). The radiographic and ultrasonographic evaluation of enteric duplication cysts. *Pediatric Radiology*.

[4] Dombale V, Patil BV, Kadam SA, Kerudi BH (2012). Enteric duplication cyst of caecum presenting with intestinal obstruction—a case report. *Journal of Krishna Institute of Medical Sciences University*.

[5] Tong SC, Pitman M, Anupindi SA (2002). Best cases from the AFIP: ileocecal enteric duplication cyst: radiologic-pathologic correlation. *Radiographics*.

[6] Choi SO, Park WH, Kim SP (1993). Enteric duplications in children—an analysis of 6 cases. *Journal of Korean Medical Science*.

[7] Prada Arias M, García Lorenzo F, Montero Sánchez M, Muguerza Vellibre R (2006). Enteric duplication cyst resembling umbilical cord cyst. *Journal of Perinatology*.

[8] Otter MI, Marks CG, Cook MG (1996). An unusual presentation of intestinal duplication with a literature review. *Digestive Diseases and Sciences*.

[9] Shah A, Du J, Sun Y, Cao D (2012). Dynamic change of intestinal duplication in an adult patient: a case report and literature review. *Case Reports in Medicine*.

[10] Blank G, Königsrainer A, Sipos B, Ladurner R (2012). Adenocarcinoma arising in a cystic duplication of the small bowel: case report and review of literature. *World Journal of Surgical Oncology*.

[11] Bremer JL (1944). Diverticula and duplications of the intestinal tract. *Archives of Pathology & Laboratory Medicine*.

[12] Fotiadis C, Genetzakis M, Papandreou I, Misiakos EP, Agapitos E, Zografos GC (2005). Colonic duplication in adults: report of two cases presenting with rectal bleeding. *World Journal of Gastroenterology*.

[13] Royal SA, Hedlund GL, Kelly DR (1994). Ileal duplication cyst. *The American Journal of Roentgenology*.

[14] Johnson JA, Poole GV (1994). Ileal duplications in adults: presentation and treatment. *Archives of Surgery*.

[15] Zhang Z, Jin F, Wu H, Tan S, Tian Z, Cui Y (2013). Double esophageal duplication cysts, with ectopic gastric mucosa: a case report. *Journal of Cardiothoracic Surgery*.

[16] Barr LL, Hayden CK, Stansberry SD, Swischuk LE (1990). Enteric duplication cysts in children: are their ultrasonographic wall characteristics diagnostic?. *Pediatric Radiology*.

[17] Cheng G, Soboleski D, Daneman A, Poenaru D, Hurlbut D (2005). Sonographic pitfalls in the diagnosis of enteric duplication cysts. *American Journal of Roentgenology*.

[18] Srivastava P, Gangopadhyay AN, kumar V (2009). Noncommunicating isolated enteric duplication cyst in childhood. *Journal of Pediatric Surgery*.

[19] Holcomb GW, Gheissari A, O'Neill JA, Shorter NA, Bishop HC (1989). Surgical management of alimentary tract duplications. *Annals of Surgery*.

